# The Hepatoprotective Properties of Gentiopicroside, Sweroside, and Swertiamarin Against Metabolic Dysfunction-Associated Steatotic Liver Disease (MASLD) †

**DOI:** 10.3390/biom15050726

**Published:** 2025-05-16

**Authors:** Anthony O. Boateng, Vinood B. Patel, S. W. Annie Bligh

**Affiliations:** 1 School of Science, Faculty of Engineering & Science, University of Greenwich, Central Avenue, Chatham, Kent ME4 4TB, UK; 2 School of Life Science, College of Liberal Arts and Science, University of Westminster, 115 New Cavendish Street, London W1W 6UW, UK; 3 S. K. Yee School of Health Sciences, Saint Francis University, Hong Kong, China

**Keywords:** gentiopicroside, sweroside, swertiamarin, silymarin, hepatoprotective, phytochemicals

## Abstract

Metabolic dysfunction-associated steatotic liver disease (MASLD) is a metabolic disease characterised by the accumulation of fat in the liver. It is estimated that 30–38% of the world’s adult population have MASLD, making it the most prevalent global chronic liver disease. Due to a lack of a therapy for MASLD, treatment has been mainly focussed on managing the conditions associated with the disease such as obesity, diabetes mellitus, and hyperlipidaemia. This study aimed to investigate the role played by *Gentiana* phytochemicals including the following: gentiopicroside, sweroside, and swertiamarin, in promoting hepatocyte protection against the cytotoxic effects of fatty acids. *Gentiana* species such as lutea, macrophylla, rigescens, and scabra are known to protect and enhance hepatocyte viability via their antioxidant, anti-inflammatory, and bitter components including the following: amarogentin gentianine, iso-orientin, swertiamarin, gentiopicroside, and sweroside. In this study, HepG2 cells pre-treated with phytochemicals gentiopicroside, sweroside, swertiamarin, and silymarin followed by an exposure to arachidonic acid (10, 30, 50 and 80 µM) were assessed for cell viability via MTT, mitochondrial function via seahorse assay, ROS levels via DCF assay, and annexin V-FITC for apoptosis. THLE-2 cells were also assayed for validation. The phytochemicals tested improved ATP production notably gentiopicroside, which improved ATP production by over 60% compared to untreated hepatocytes. Significant hepatocyte protection against lipotoxicity leading to apoptosis was also observed in gentiopicroside in the presence of 30 µM arachidonic acid with apoptosis reduced by over 50%. ROS production was reduced up to 60% by the pre-treatment of HepG2 cells with 20 µM, gentiopicroside, sweroside, swertiamarin, and silymarin, with the highest reduction observed in swertiamarin. It was concluded that phytochemicals gentiopicroside, sweroside, and swertiamarin play key roles in the hepatocyte protection against the cytotoxic effects of fatty acids. This protection is conferred by enhancing mitochondrial function in terms of increasing the maximal respiratory capacity in response to a high influx of fatty acids, promoting ATP production as well as scavenging ROS produced as a result of high fatty acid influx and increased mitochondrial respiration. Highlights: Gentiopicroside may minimise lipotoxicity leading to apoptosis and necrosis in hepatocytes in the presence of arachidonic acid. A pre-treatment of hepatocytes with phytochemicals, namely gentiopicroside, sweroside, and silymarin provides a degree of protection which may be attributed to the enhancement of mitochondrial function. Sweroside, silymarin, and swertiamarin may protect HepG2 and THLE-2 cells by scavenging ROS produced by arachidonic acid and the mitochondrial electron transport chain.

## 1. Introduction

MASLD comprises isolated hepatic steatosis, metabolic dysfunction-associated steatohepatitis (MASH), and liver fibrosis, eventually leading to cirrhosis. Beyond the liver, the effects of MASH can impact multiple systems within the body leading to chronic kidney disease [1,2,3]. The underlying factors associated with the pathogenesis of MASLD includes the elevation of plasma fatty acids and glucose concentration coupled with insulin resistance derived from hepatic fat accumulation. These factors result in elevated fatty acid synthesis coupled with diminished beta-oxidation, eventually leading to MASLD onset. Evidence shows that increased beta-oxidation as a result of downregulating MARC1 can reduce the amount of lipids in hepatocytes [4,5,6]. The increased accumulation of fatty acids in hepatocytes is a direct cause of mitochondrial damage and oxidative stress due to cathepsin B activation in response to lysosomal damage caused by fatty acid accumulation [7,8]. Oxinflamation, a term illustrating the relationship between oxidative stress and metabolic inflammation, can also be triggered by mitochondrial dysfunction and lipotoxicity, eventually leading to insulin resistance. Due to the link between fatty acid-induced lipotoxicity and increased mitochondrial damage, a reduction in mitochondrial damage has the capacity to mitigate the effects of fatty acid lipotoxicity [9,10].

Studies have shown that arachidonic acid causes apoptosis by producing cytosolic phospholipase A_2_, eventually causing mitochondrial permeability transition [11,12].

In another study, gentiopicroside, sweroside, and swertiamarin were responsible for hepatoprotective effects exerted by *Gentiana manshurica* as well as *Gentiana turkestanerum* against carbon tetrachloride-induced hepatic damage in mice [13]. Gentiopicroside, sweroside, and swertiamarin found in *Gentiana scabra* exerted hepatoprotective effects on hepatocytes by diminishing oxidative stress [14,15], whereas in a rat liver toxicity model, swertiamarin showed hepatoprotective effects by significantly reducing liver injury [16]. In a rat liver damage model induced by α-naphthylisot hiocyanate, swertiamarin at a dose of 20 mg/kg portrayed hepatoprotective effects by significantly reducing alanine aminotransferase, aspartate aminotransferase, and the total and direct bilirubin levels which had been increased in the presence of α-naphthylisot hiocyanate while conversely increasing bile flow [16]. *Gentiana lutea* extracts reduced lipid peroxidation, protein carbonylation, oxidative stress, and the overall hepatotoxicity in rat models with ketogonazole-induced oxidative stress [17]. *Gentiana rigescens* contain a key phytochemical (gentiopicroside) which significantly reduced alpha-naphthylisothiocyanate (75 mg/kg, ig)-induced hepatic injury in rats by upregulating hepatic mRNA levels of synthesis enzymes (Cyp8b1 and Cyp27a1), transporters (Mrp4 Mdr1 and Ost-β), and ileal bile acid circulation mediators (Asbt and Fgf15) [18]. Antioxidant phytochemicals including sweroside, swertiamarin, and gentiopicroside found in swertia *chirayita* extract may be responsible for hepatoptotective action in HepG2 cells exposed to acetaminophen [19]

Phytochemicals gentiopicroside, sweroside, and swertiamarin found in *Gentiana* species were studied with the aim of determining whether or not they conferred hepatocyte protection in the presence of arachidonic acid. The study entailed an initial determination of their individual effects on hepatocytes (HepG2 and THLE-2) cell viability in the presence of fatty acids via MTT assay. This was followed by a sequence of assays including the seahorse mitochondrial stress assay with a focus on hepatocyte ATP production, non-mitochondrial respiration, proton leak, basal respiration, maximal respiration, and spare respiratory capacity, in the presence of oligomycin, FCCP, antimycin, and rotenone. The study was finalised by conducting DCF ROS and annexin V-FITC apoptosis flow cytometric assays. These phytochemicals were studied jointly with silymarin which is a well-known hepatoprotective phytochemical derived from milk thistle (*Silybum marianum*). Silymarin has been shown to possess antioxidant and hepatoprotective protective properties [20,21,22,23].

## 2. Materials and Methods

### 2.1. Cell Culturing

Human hepatocellular (HepG2) cells and THLE-2 cells were obtained from (ATTC, London, UK). HepG2 were cultured in Dulbecco’s modified eagle media (DMEM) with 4 g/L glucose (Lonza, Slough, UK) supplemented with foetal bovine serum (FBS) 10% (Biosera, Sussex, UK), sodium pyruvate 1% (Sigma-Aldrich, London, UK), L-glutamine 1% (Sigma-Aldrich, London, UK), and penicillin-streptomycin 1% (BioWest, Lakewood, FL, USA).

### 2.2. Phytochemicals and Arachidonic Acid Preparation

Gentiopicroside (Abcam, Cambridge, UK), sweroside (Sigma-Aldrich, London, UK), swertiamarin (Sigma-Aldrich, London, UK), and silymarin (Abcam, Cambridge, UK) were prepared by making 8 mM stock solutions in DMSO and then diluting them with DMEM containing FBS 10% to obtain a 20 µM final concentration. An 8 mM stock of arachidonic acid was prepared in DMSO and diluted to 10, 30, 50, and 80 µM with DMEM as per the requirements of each assay.

### 2.3. MTT Assay for Measuring the Cell Viability of Cells Pre-Treated with Phytochemicals Gentiopicroside, Sweroside, and Silymarin in the Presence of Arachidonic Acid

HepG2/THLE-2 cells were trypsinised and seeded at a concentration of 25 × 10^3^/200 μL DMEM per well for 24 h. The media was then removed, and three different types of phytochemical treatments were applied for 24 h, after which the cells were exposed to arachidonic acid of 10, 30, 50, and 80 µM. An MTT assay was performed after 24 h by removing the treatments/media and replacing it with 90 μL of media. Thiazole blue tetrazolium bromide (TBT) (Sigma-Aldrich, London, UK) 10 μL containing 5 mg/mL TBT in PBS was added per well and incubated at 37 °C for 2 h. Following removal, 50 μL DMSO was added per well. The plates were read at 550 nm after being incubated at room temperature for 15 min. Cell viability was presented as a percentage of control cells with DMSO.

### 2.4. The Seahorse Assay for Assessing the Mitochondrial Function of Cells Pre-Treated with Gentiana Species and Phytochemicals in the Presence of Arachidonic Acid

A seahorse assay was performed by seeding HepG2/THLE-2 cells in seahorse XF24 plates at a concentration of 5 × 10^3^/250 μL DMEM per well and kept for 24 h in an incubator (Binder APT, Tuttlingen, Germany) at 37 °C. The media was removed and the cells were pre-treated with single compounds: (gentiopicroside, silymarin, swertiamarin or sweroside), of a total of 20 µM, and incubated for another 24 h at 37 °C. The media containing treatment was discarded after the incubation period and replaced with media containing 30 μM AA and then incubated at 37 °C for 24 h. After incubation, the seahorse assay was initiated by removing the media and washing thrice with 400 μL of seahorse media containing 1% sodium pyruvate and 4.4 g/L glucose, and the media stabilised at pH 7.4. After washing, 500 μL of seahorse media was placed in each well and then incubated in a non-CO_2_ incubator (to minimalise the influence of the incubation conditions) pending the completion of the calibration plate running. The calibration plate was prepared by placing oligomycin (5 μM), FCCP (5 μM), antimycin, and rotenone (5 μM), after which it was placed in the seahorse XFe 24 analyser (Aglient/Seahorse Bioscience, Santa Clara, CA, USA). After calibration, the assay plate was removed from the non-CO_2_ incubator and placed in the seahorse XFe 24 which measured the oxygen consumption rate (OCR) in pmol/min at oligomycin, FCCP, antimycin, and rotenone injection points. The hepatocytes in the plate were normalised to protein via the BCA protein assay. Taking normalisation results, basal respiration, ATP production, proton leak, maximal respiration, spare respiratory capacity, and non-mitochondrial respiration were calculated.

### 2.5. The DCF Assay for Assessing the ROS Produced by Cells Pre-Treated with Gentian spp. and Single Compounds: Gentiopicroside, Sweroside, Swertiamarin, and Silymarin in the Presence of Arachidonic Acid

HepG2 cells were seeded to make available 1.5 × 10^5^ cells per well in dark, clear-bottom 96-well plates optimised for fluorescence-based applications (Thermo Fisher Scientific, UK). After 24 h, a arachidonic treatment and DCF assay was performed by washing each well with 100 µL of 1× buffer supplied with a DCFDA-cellular reactive oxygen species detection assay kit (Abcam, UK). As a positive control, HepG2 cells were treated with tert-butyl hydrogen peroxide (TBHP) 50 µM for 2 h. This treatment, as well as the 100 µL of 1× buffer, were removed and DCFDA assay reagent 100 µL of 20 µM was added to each well and incubated for 30 min at a temperature of 37 °C away from light. DCFDA was then removed from each well and replaced with 100 µL of 1× buffer followed by the measurement of fluorescence with (Fluostar Optima, BMG Labtech, UK) at excitation 485 nm and emission 535 nm.

### 2.6. An Annexin V-FITC PI Assay for Investigating Apoptosis in Hepatocytes Pre-Treated with Gentiana Macrophylla and Single Compounds: Gentiopicroside, Prior to Arachidonic Acid Exposure

HepG2 cells were seeded overnight in a 12-well plate at a concentration of 20 × 10^4^ cells/mL DMEM per well for 24 h and kept in an incubator at 37 °C. The media was then removed after which single compound gentiopicroside (20 µM) and *Gentiana macrophylla* (10 µg/mL) pre-treatments were applied and incubated at 37 °C for 24 h and then the treatment was removed and replaced with arachidonic acid (30 μM) and incubated again for 24 h. Apoptosis was induced in the positive control group by adding 1 µg/mL actinomycin whereas the negative control had cells with DMEM without any apoptosis inducing agent. Cells were harvested and washed in cold phosphate-buffered saline (PBS), re-centrifuged, and then re-suspended in 100 µL of 1× binding buffer after discarding the supernatant. Annexin V-FITC (5 µL) and propidium iodide (PI) 5 µL (Stratech, UK) were added to each 100 µL of cell suspension. The cells were then incubated at room temperature for 15 min followed by the addition of 400 µL of 1× buffer. Flow cytometric measurements of the samples at a fluorescence of 530 nm (emission) and 575 nm were taken. Apoptotic cells showed a green fluorescence whereas necrotic cells showed both a red and green fluorescence.

### 2.7. Statistics

Results refer to the mean ± standard deviation and are average values from n = 3–7 values per experiment. The evaluation of hepatocyte protection conferred by single compounds at different concentrations of AA was performed via the two-way ANOVA with a Tukey multiple comparison test. Differences at *p* < 0.05 were considered significant.

## 3. Results

### 3.1. A Comparison of the Cytotoxic Effects of Fatty Acid on Single Compounds: Gentiopicroside, Sweroside, and Silymarin Pre-Treated Hepatocytes (HepG2)

**Figure 1:**
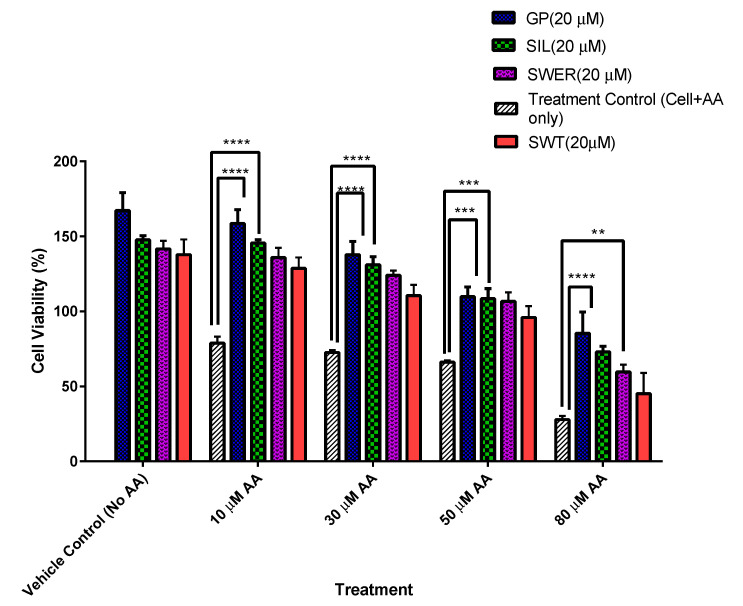
4,5-dimethylthiazol-2-yl (MTT) assay results showing the hepatocyte protection conferred by phytochemicals. 4,5-dimethylthiazol-2-yl (MTT) assay results showing the hepatocyte protection conferred by the gentiopicroside (GP), silymarin (SIL), swertiamarin (SWT), and sweroside (SWER) pre-treatment for 24 h. For all phytochemical pre-treated hepatocytes (HepG2), AA cytotoxicity decreased compared with untreated cells. Gentiopicroside (GP)-treated hepatocytes presented the highest viabilities (85–159%) in the presence of arachidonic acid (AA) (10–80 μM). The two-way ANOVA with the Tukey multiple comparison of the phytochemical treatment factor and control (** *p* = 0.0060), (*** *p* = 0.0002), and (**** *p* < 0.0001).

This study investigated whether pre-treating cells with gentiopicroside, sweroside, and silymarin prior to fatty acid exposure conferred a degree of hepatocyte protection to the cells. In order to establish this, HepG2 cells were treated with the above-listed compounds (20 μM) for 24 h, after which the treatment was replaced with media containing AA (10, 30, 50, and 80 μM) for another 24 h (Figure 1). Cell viability was then studied via an MTT assay. The consistency in reduced AA cytotoxicity was observed in all pre-treated hepatocytes with percentage viabilities ranging from (60–159%). Hepatocytes pre-treated with gentiopicroside had the highest range of cell viability (85–159%) across all doses of fatty acid exposure compared to untreated hepatocytes. This was followed by silymarin with a range of (73–145%) and then sweroside with a range of (60–135%). Vehicle control cells which had been not exposed to any arachidonic after phytochemical pre-treatment had the highest viabilities recorded for each treatment. The lowest cell viability of 28% was recorded for hepatocytes exposed to arachidonic acid without any phytochemical pre-treatment.

### 3.2. A Comparison of the Cytotoxic Effects of Fatty Acid on Single Compounds: Gentiopicroside, Sweroside, and Silymarin Pre-Treated THLE-2 Cells (THLE-2)

**Figure 2:**
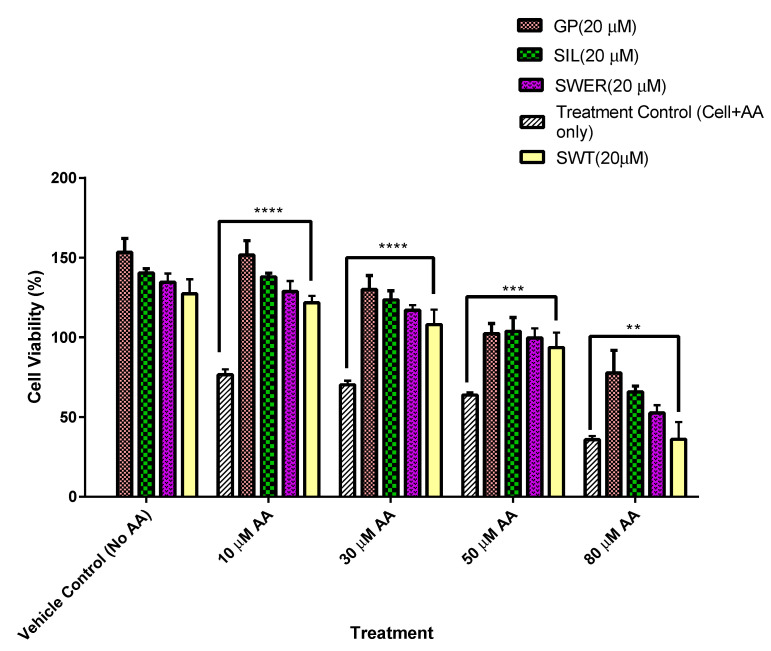
Hepatocyte protection conferred on transformed human liver epithelial -2 (THLE-2) cells by phytochemical pre-treatment for 24 h. For all phytochemical pre-treated hepatocytes, the arachidonic acid (AA) cytotoxicity decreased compared with untreated cells. Gentiopicroside (GP)-treated hepatocytes presented the highest viabilities (77–153%) in the presence of arachidonic acid (AA) (10–80 μM). Hepatocytes treated with only phytochemicals (i.e., vehicle control) yielded viabilities up to 153%. The data are presented as the mean ± SD two-way ANOVA with Tukey multiple comparisons **** *p* = 0.0001, *** *p* = 0.0003, and ** *p* = 0.001.

In a similar manner to HepG2 cells, THLE-2 cells treated with phytochemicals gentiopicroside, sweroside, and swertiamarin showed reduced AA cytotoxic effects in terms of diminished cell viability compared to control cells which had not been primed with phytochemicals. Using THLE-2 cells helped to determine if the cell growth enhancement was only limited to HepG2 cells or could be seen in other cell types such as THLE-2 cells which are hepatocytes transformed with SV40 large T antigen. Cell viability was within the range of 77 to 153% for gentiopicroside which elicited the highest hepatocyte viability among the phytochemicals tested when compared to the control. There was a general trend of cell viability reducing with an increase in AA concentration. Cells which were devoid of priming with phytochemicals but exposed to AA (10–80 μM) yielded viabilities of 35–76%. Other phytochemicals including sweroside, swertiamarin, and silymarin enhanced cellular viability as well by up to 137%. The treatment of hepatocytes with phytochemicals alone did not appear to diminish the cell viability of hepatocytes but rather enhanced it with viabilities of 127, 134, 140, and 153% recorded for swertiamarin, sweroside, silymarin, and gentiopicroside, respectively, as shown in Figure 2.

### 3.3. A Comparison of the Effects of Single Compounds: Gentiopicroside, Sweroside, and Silymarin Pre-Treatment on Hepatocyte Mitochondrial Function in the Presence of Arachidonic Acid

**Figure 3:**
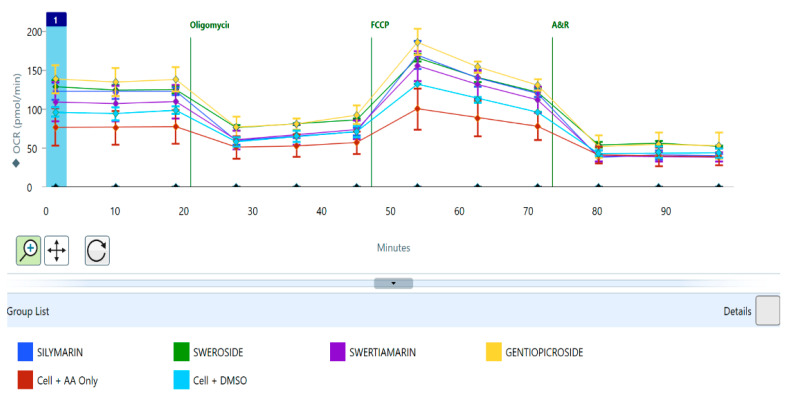
Typical seahorse mitochondrial stress test trace for phytochemicals. Typical seahorse mitochondrial stress test trace for gentiopicroside, silymarin, swertiamarin, and sweroside showing the injection points of oligomycin, carbonyl cyanide-p-trifluoromethoxyphenylhydrazone (FCCP), antimycin, and rotenone and the resultant effect on the oxygen consumption rate (OCR) of hepatocytes after injection with GPS pre-treated hepatocytes exhibiting the highest OCRs.

**Figure 4:**
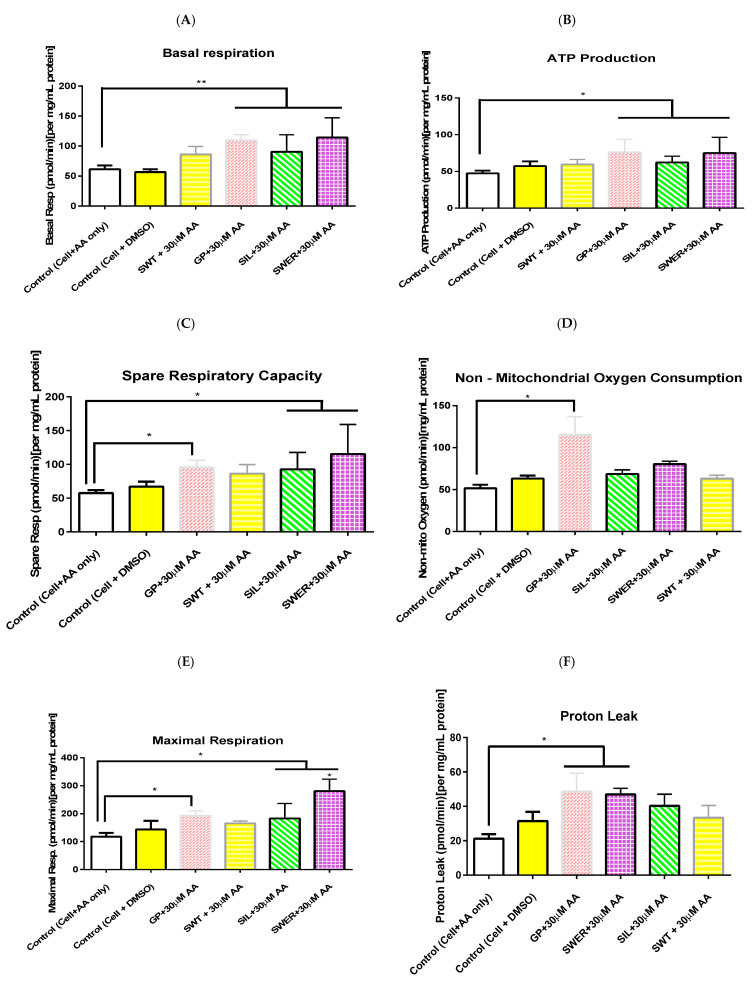
Seahorse mitochondrial stress test of gentiopicroside, sweroside, and swertiamarin. Seahorse mitochondrial stress test of gentiopicroside, sweroside, and swertiamarin showing the following: (**A**) the basal respiration in pre-treated hepatocytes up to 114 pmol/min ** *p* = 0.0055; (**B**) the ATP production in pre-treated hepatocytes up to 75.9 pmol/min * *p* < 0.05; (**C**) the spare respiratory capacity in pre-treated hepatocytes up to 115.2 pmol * *p* < 0.05; (**D**) the non-mitochondrial respiration in pre-treated hepatocytes up to 114.9 pmol * *p* < 0.05; (**E**) the maximal respiration in pre-treated hepatocytes up to 281 pmol * *p* < 0.05; (**F**) the proton leak up to 48 pmol * *p* < 0.05. All data were analysed via two-way ANOVA assessing the significance of phytochemical treatment. A general trend of improved efficiency of mitochondrial function parameters in pre-treated HepG2 cells was seen.

The seahorse mitochondrial stress test enabled the measurement of basal respiration, ATP production, proton leak, maximal respiration, spare respiratory capacity, and non-mitochondrial respiration in hepatocytes pre-treated with gentiopicroside, sweroside, swertiamarin, and silymarin (20 μM) before being exposed to arachidonic acid (30 μM). A typical seahorse trace for gentiopicroside, sweroside, and swertiamarin is shown in Figure 3. The concentration of ATP produced by phytochemical pre-treated hepatocytes appeared to increase compared to untreated hepatocytes exposed to arachidonic acid (Figure 4B). Gentiopicroside pre-treated hepatocytes caused an ATP production of 75.9 pmol/min followed by sweroside with 75 pmol/min. Basal respiration was also enhanced in pre-treated hepatocytes compared to untreated hepatocytes exposed to fatty acids (Figure 4A). Sweroside pre-treated hepatocytes presented the highest basal respiration of 114 pmol/min followed by gentiopicroside with 109 pmol/min. Pre-treating hepatocytes with phytochemicals also enhanced the maximal respiratory capacity of the cells even after they were exposed to arachidonic acid (Figure 4E). This effect was mostly seen with the sweroside pre-treatment up to 281 pmol/min followed by gentiopicroside up to 192 pmol/min. Gentiopicroside pre-treated hepatocytes presented the highest non-mitochondrial respiration of 115 pmol/min followed by sweroside with 80 pmol/min (Figure 4D). The spare respiratory capacity of hepatocytes was markedly increased by sweroside up to 115 pmol/min followed by gentiopicroside up to 95 pmol/min (Figure 4C). As far as proton leak is concerned, it was observed in all the phytochemicals used, but markedly seen in gentiopicroside and followed by sweroside up to 49 pmol/min to (202, 77, 76, and 52 pmol/min), respectively (Figure 4F). In the case of control cells with DMSO as well as negative control cells with only AA treatment, reduced OCR rates were recorded for all the parameters studied.

### 3.4. The Effect of Phytochemicals Gentiopicroside, Sweroside, Swertiamarin, and Silymarin Pre-Treatment on Hepatocyte ROS Production in the Presence of Arachidonic Acid

**Figure 5:**
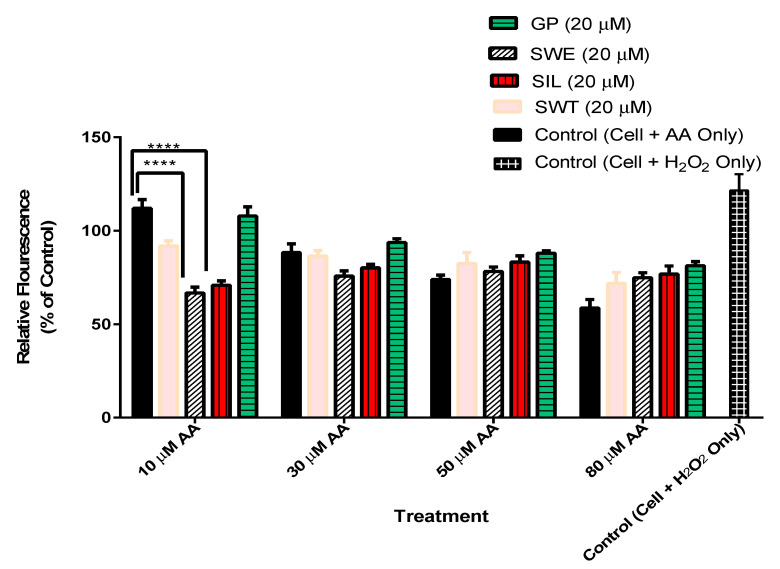
2,7-dichlorofluorescein (DCF) assay results of HepG2 cells exposed to AA. Results of the DCF assay showing relative fluorescence which depicts the amount of reactive oxygen species (ROS) produced at each instant. The reactive oxygen species (ROS) is scavenged to a degree by pre-treatments but is markedly so in sweroside and silymarin (67 and 71%), respectively. Higher doses of arachidonic acid (AA) (30, 50, and 80 µM) show a decrease in the amount of ROS produced. The results were analysed by two-way ANOVA with Tukey multiple comparisons **** *p* < 0.0001.

This test evaluated the ROS scavenging effects of the above-listed phytochemicals in comparison to silymarin which is a well-known ROS scavenging phytochemical. In this instance, the presence of AA (10 µM) caused an increase in ROS by up to 112% which, however, decreased at higher doses of AA (30, 50, and 80 µM) (Figure 5). Although there were variations in the amounts of ROS scavenged by the different pre-treatments, sweroside and silymarin were the most consistent and portrayed the best ROS scavenging capacity of up to 67 and 71%, respectively (Figure 5).

### 3.5. A Comparative Assessment of Hepatocyte (HepG2) Protection via Apoptosis and Necrosis Prevention by Gentiana Macrophylla and Gentiopicroside

**Figure 6:**
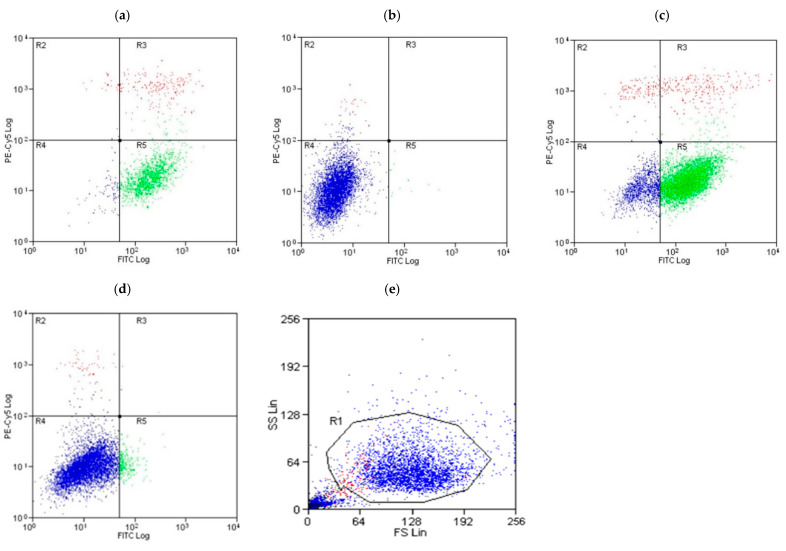
Results of the Annexin V-FITC and PI assay. (**a**) Scatter diagrams of positive control cells exposed to 1 µg/mL actinomycin showing a high level of apoptosis. (**b**) Negative control cells seeded with Dulbecco’s modified eagle medium (DMEM) and 0.1% dimethylsulfoxide (DMSO) only showing a high proportion of live cells. (**c**) Cells with 30 μM arachidonic acid (AA) only and no drug pre-treatment presenting live, apoptotic, and necrotic cells. (**d**) Cells with the 20 μM gentiopicroside (GP) pre-treatment for 24 h before 30 μM arachidonic acid (AA) exposure showing a high proportion of live cells and (**e**) flow cytometry gating strategy.

**Figure 7:**
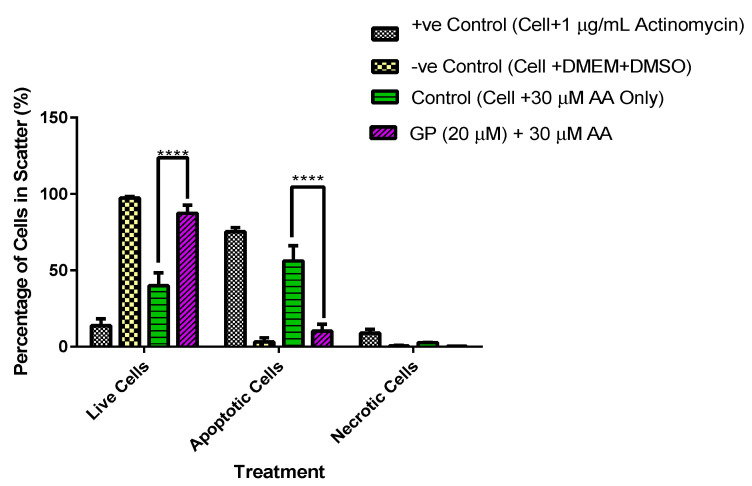
A histogram showing the level of apoptosis and necrosis in hepatocytes pre-treated with GP. Apoptosis is reduced by up to 50% in gentiopicroside (GP) pre-treated hepatocytes compared to control cells without any pre-treatment prior to AA exposure. Two-way ANOVA with a Tukey multiple comparison of the data shows a statistically significant difference between GP pre-treated cells and control cells exposed to AA without any drug pre-treatment **** *p* < 0.0001.

The annexin V-FITC-PI assay was used to assess whether or not pre-treating hepatocytes with gentiopicroside prevented apoptosis and necrosis in the presence of 30 µM arachidonic acid. Scatter diagrams of the results showed a high degree of apoptosis (75%) and low necrosis (9%) in positive control cells exposed to 1 µg/mL actinomycin (Figure 6a and Figure 7). Negative control cells seeded with DMEM and DMSO 0.1% only also showed a high proportion of live cells (97%) (Figure 6b and Figure 7). The treatment of hepatocytes with 30 µM AA increased apoptosis up to 56% as seen in Figure 6c and Figure 7. The pre-treatment of hepatocytes with gentiopicroside prior to arachidonic acid exposure increased the proportion of live cells up to 95% while reducing apoptosis to 3.1% as seen in Figure 6d,e and Figure 7. Necrosis was also reduced significantly in the presence of the gentiopicroside pre-treatment.

## 4. Discussion

This study aimed to establish whether phytochemicals gentiopicroside, sweroside, and swertiamarin provided hepatocyte protection against fatty acid-induced hepatic injury in comparison with silymarin. It was generally observed that phytochemicals gentiopicroside, sweroside, and swertiamarin conferred hepatocyte protection in terms of enhancing cell growth by promoting mitochondrial function in the presence of AA, preventing apoptosis and the build-up of ROS. These effects were in some cases greater than those elicited by silymarin. The choice of the 20 µM phytochemicals and 24 h timeframe followed observations in studies such as [24] where 20 µM gentiopicroside reduced lipid accumulation, and promoted glucose consumption and glycogen storage in pre-treated HepG2 cells exposed to palmitic acid for 24 h.

Furthermore, studies by [25] indicated that 20 µM of silymarin had significant effects on in vitro assays with CypExpress™ Cytochrome P450 human kits. With these baselines, the dose of 20 µM was chosen as a benchmark to compare phytochemicals in this study. In addition, previous studies carried out in our lab to investigate mitochondrial activity [26] entailed the exposure of HepG2 cells to arachidonic acid for a 24 h period.

Gentiopicroside pre-treated hepatocytes emerged with the highest viability, followed by silymarin, sweroside, and finally swertiamarin in order of decreasing cell viability in HepG2 cells. The oral administration of gentiopicroside 50 mg/kg to fat-fed mice caused a significant reduction in the expression of adepogenic factors (PPARγ, C/EBPα, and SREBP-1c) while inhibiting lipid and triglyceride uptake genes [27]. The administration of gentiopicroside 40–80 mg/kg to mice prior to a lipopolysaccharide injection of 10 μg/kg demonstrated hepatoprotective activity by significantly reducing serum aminotransferase, lipid peroxidation, and TNF-α activity [28]. This result further agreed with studies by [29] showing that after pre-treating chondrocytes with 50–150 µg/mL of gentiopicroside for 24 h followed by MTT, there was no toxic effects present but rather increased function. Gentiopicroside is known to possess hepatoprotective effects on d-galactosamine and lipopolysaccharide-induced hepatic failure [28]. Furthermore, gentiopicroside was shown to exhibit hepatoprotective effects on the IL-1β-induced inflammation response in rat articular chondrocyte. Sweroside, which was the third most effective phytochemical in terms of cell viability maintenance in this study, has shown hepatoprotective properties against carbon-tetrachloride-induced injury in rats [30]. It was, however, observed that cell viability enhancement was more pronounced in HepG2 cells than THLE-2 cells. This could be because HepG2 cells possess a higher sensitivity for basic compounds whereas THLE-2 cells possessed a higher sensitivity for acidic and neutral compounds [31].

Mitochondrial function was assessed in terms of ATP production, basal respiration, maximal respiration, spare respiratory capacity, proton leak, and non-mitochondrial respiration in pre-treated (i.e., primed) hepatocytes via the seahorse mitochondrial stress test, since impaired mitochondrial respiration and hepatic ATP synthesis has been associated with the accumulation of fatty acids in hepatocytes [32]. Hepatocytes pre-treated with gentiopicroside, silymarin, sweroside, and swertiamarin showed a higher rate of basal respiration and ATP production of up to 75 pmol/min observed with gentiopicroside which was the highest compared to sweroside, swertiamarin, and silymarin.

These results denote the possibility that phytochemicals gentiopicroside, sweroside, and swertiamarin may protect hepatocytes from arachidonic acid-induced cytotoxicity by enhancing mitochondrial function in terms of ATP production, basal respiration of cells, increasing cellular respiratory capacity as seen in maximal respiration results, and also in terms of broadening the spare respiratory capacity of hepatocytes which is required to meet the rapid energy demands of the cells especially for dealing with a high influx of fatty acids (AA).

Gentiopicroside pre-treated cells had a very high non-mitochondrial respiration capacity of 115 pmol/min, raising the possibility that the effects of gentiopicroside on hepatocytes extend beyond the mitochondria into other cellular organelles. This, however, needs to be confirmed through further investigations. There is evidence which indicates that hepatic mitochondrial dysfunction is crucial to the pathogenesis of MASLD. This is because the resultant electron flow disruption associated with a dysfunctional mitochondrial respiration causes the preceding respiratory intermediates to transfer electrons to molecular oxygen, hence producing superoxide anions and hydrogen peroxide in the process [33]. Hence, the protection and enhanced function conferred by gentiopicroside, sweroside, and swertiamarin to the mitochondria could be a point of intervention in the pathogenesis of MASLD.

Proton leak is one key factor which affects mitochondrial coupling efficiency and ROS production. It is cell-type specific, caused by mitochondrial anion carriers directly proportional to cellular metabolic rate [34]. This correlation between proton leak and cellular metabolic rate may have contributed to the increased amount of proton leak observed in pre-treated HepG2 as seen in the seahorse results. The phytochemicals gentiopicroside and sweroside which produced the highest ATP productions also observed an increased proton leak. The site for proton leak is in the inner mitochondrial membrane of eukaryotes and accounts for about 20% of standard metabolic rates in rats [35]. As a result, lower levels of proton leak of up to 21 pmol/min was observed for control cells even though they had no phytochemical pre-treatment.

Mitochondria serve as a major intracellular source of ROS generated at complex I and III of the respiratory chain. It was observed that upon treating hepatocytes with 10 µM arachidonic acid, ROS levels were increased up to 112% (Figure 5). However, this study showed that the phytochemicals (sweroside, silymarin, and swertiamarin) scavenged the produced ROS (Figure 5), where sweroside possessed the highest ROS scavenging effect, followed by silymarin and swertiamarin. Sweroside has been found to possess reactive oxygen species scavenging effects [36]. Secoiridoid glycosides inhibit free radical activity and prevent the onset of peroxidation reactions, as is explained in [37]. In HepG2 cells, silymarin showed antioxidant and hepatoprotective activity against tacrine-induced cytotoxicity [38]. A dose of 10–100 µM of silymarin possessed antioxidant effects in HepG2 cells against bleomycin, which is a known ROS generator [39]. In this study, ROS production was reduced up to 60% by the pre-treatment of HepG2 cells with 20 µM of gentipicrosie, sweroside, swertiamarin, and silymarin with the highest reduction observed in swertiamarin. At a dose of 80 µM AA, the increase in ROS production in HepG2 cells was reduced possibly as a result of the increased cell death in untreated hepatocytes due to high oxidative stress. Oxidative stress leading to cell death can be caused by an imbalance between reactive oxygen species and antioxidant defences [40]. Hence, the lack of an active ROS scavenger can be detrimental to the viability of hepatocytes exposed to ROS-producing compounds.

As seen in the seahorse mito stress assay, gentiopicroside and sweroside acted on mitochondrial complex I and III, producing a very high basal respiration, but sweroside pre-treated cells, apart from having a high basal respiration, had the highest maximal respiration capacity whereas gentiopicroside pre-treated cells had a low maximal respiration capacity. This may account for the better performance of sweroside than gentiopicroside in managing ROS generated by hepatocytes.

Loss of cell function and eventual apoptosis or necrosis are the end results of oxidative stress emanating from high ROS levels [41]. The annexin V-FITC-PI assay assessed the anti-apoptotic/anti-necrotic effect of the gentiopicroside pre-treatment against arachidonic acid-induced apoptosis/necrosis. The presence of polyunsaturated fatty acids such as arachidonic acid coupled with the increased production of reactive oxygen intermediates causes cellular toxicity leading to lipid peroxidation and eventually apoptosis [42]. Arachidonic acid is also an intermediate in apoptosis signalling regulated by cytochrome c oxidase subunit 2 (COX-2) and fatty acid-CoA ligase 4 (FACL4) [43]. These studies support the increased necrosis and apoptosis observed in control cells exposed to arachidonic acid 30 µM without any gentiopicroside pre-treatment. Apoptosis was, however, markedly reduced in gentiopicroside pre-treated cells by up to 43%. As observed in this study, a diminished ATP production was observed in untreated HepG2 cells which were exposed to 30 µM of arachidonic acid. The antiapoptotic effect of gentiopicroside is credited with its hepatoprotective effects [28]. Mitochondrial dysfunction causes the release of cytochrome c and other pro-apoptotic proteins, which initiates caspase activation and apoptosis. This raises the possibility that the anti-apoptotic effect of gentiopicroside may also be linked with its ability to improve the efficiency of mitochondrial function in terms of mitochondrial ATP production and basal respiration as seen in the seahorse mito stress assay results.

## 5. Conclusions

The mitochondrion is a key organelle to MASLD pathogenesis in terms of fatty acid oxidation, mitochondrial respiration, and ATP production, as well as fatty acid synthesis. These studies have shown that pre-treating hepatocytes with phytochemicals gentiopicroside, sweroside, and silymarin provides a degree of protection which may be attributed to enhancing mitochondrial function in terms of ATP production, basal respiration, spare respiratory capacity, maximal respiration, proton leak, and non-mitochondrial oxygen consumption. Gentiopicroside showed the most promise, which upon further assessment exhibited anti-apoptotic and anti-necrotic activity in the presence of arachidonic acid. It has also been observed that apart from enhancing mitochondrial function, the phytochemicals, most notably sweroside, silymarin, and swertiamarin, protected HepG2 cells by scavenging ROS produced by arachidonic acid and the mitochondrial electron transport chain. These investigations have also pointed to the possibility of a synergistic action which could elevate hepatocyte protection if an appropriate combination of the phytochemicals studied is established. This, however, requires further investigation to quantify the scope of the protection derived from combinations of different quantities of phytochemicals.

## 6. Study Limitations

A limitation of this study was the use of the one timepoint of 24 h. Other timepoints should be explored to cover this limitation. Another limitation was the main consideration of mitochondrial function. Other organelles and pathways should be investigated as well, especially those linked with mitochondrial injury such as endoplasmic reticulum stress. There was a consideration of reactive oxygen species. However, mitoSox assay should be conducted to study superoxides within the mitochondria. This study focused only on arachidonic acid; however, other fatty acids and pathways in fatty acid regulation should be investigated to see if these can modulate fatty acid production. Finally, this study missed cytochrome C measurement in the apoptosis pathway and should investigate the effects of whole plant extracts alongside phytochemicals. In accordance with mitochondrial respiration, ATP levels (Figure 4B) were not significantly reduced which in part explains the limited amount of cells undergoing necrosis. With longer exposure times or higher concentrations of fatty acids, it is possible that more cells would undergo necrosis.

## Data Availability

Data are contained within the article.

## Abbreviations

MASLD—Metabolic Dysfunction-Associated Steatotic Liver Disease, MTT-(4,5-dimethylthiazol-2-yl)-2,5-diphenyltetrazolium bromide, ROS-reactive oxygen species, DCF—dichlorodihydrofluorescein, TBT—thiazole blue tetrazolium bromide.
